# Post-cesarean analgesia: transversalis fascia plane block versus wound infiltration in a randomized trial

**DOI:** 10.1590/1806-9282.20260662

**Published:** 2026-10-16

**Authors:** Gülşen Cebecik Teomete, Ecem Güçlü Öztürk, Irmak Çimenoğlu, Merve Ergenç, Beliz Bilgili

**Affiliations:** 1 Marmara University, Pendik Training and Research Hospital, Department of Anesthesiology and Reanimation – Istanbul, Turkey.; 2 Marmara University, School of Medicine, Department of Anesthesiology and Reanimation – Istanbul, Turkey.

**Keywords:** Cesarean section, Pain, postoperative, Nerve block, Ultrasonography, Analgesics, opioid

## Abstract

**OBJECTIVE:**

The aim of this study was to compare ultrasound-guided transversalis fascia plane block with local wound infiltration using equivalent local anesthetic doses within a standardized multimodal analgesia protocol.

**METHODS:**

This prospective, single-blind randomized trial was conducted between May and June 2024 at a tertiary care hospital. Women with American Society of Anesthesiologists physical status II–III undergoing cesarean delivery under spinal anesthesia received either bilateral ultrasound-guided transversalis fascia plane block or local wound infiltration with the same total dose and volume of bupivacaine. All patients were managed with standardized multimodal analgesia. Patients with contraindications to the study interventions or inability to use patient-controlled analgesia were excluded. The primary outcome was 24-h opioid consumption. Secondary outcomes included pain scores, rescue analgesia use, opioid-related adverse events, and complications.

**RESULTS:**

Forty-two patients (n=21/group) were analyzed. Twenty-four-hour opioid consumption was similar between transversalis fascia plane block and local wound infiltration groups (median 100 [65–152] mg vs. 85 [65–135] mg; p=0.554). Pain scores remained low and comparable throughout 48 h. Rescue analgesia was rarely required, and no postoperative nausea, opioid-related adverse events, or procedure-related complications were observed.

**CONCLUSION:**

In a standardized multimodal analgesia setting after cesarean delivery, transversalis fascia plane block did not reduce opioid consumption or improve postoperative pain scores compared with local wound infiltration.

## INTRODUCTION

According to World Health Organization (WHO) data, cesarean section (CS) rates are increasing, currently at 21% of births and projected to reach 29% by 2030^1^. Effective postoperative analgesia is essential, particularly as cesarean section (CS) requires techniques that are safe for both mother and newborn^2^. In obstetric practice, simple and reproducible methods are especially valuable in high-volume settings. The PROcedure-SPECific Postoperative Pain ManagemenT (PROSPECT) guideline recommends local wound infiltration and/or regional techniques (e.g., transversus abdominis or quadratus lumborum blocks) with Grade A evidence for pain control^3^. A meta-analysis reported reductions in 24-h morphine consumption of 11 mg with transversalis fascia plane block (TFPB) and 8 mg with local wound infiltration (LWI)^4^.

The TFPB is a regional technique targeting the iliohypogastric and ilioinguinal nerves in their proximal course and has been proposed as an effective option for lower abdominal surgery^5,6^. Although several trials^7,8^ have evaluated transversalis fascia plane block for cesarean analgesia, reported mixed results and many comparisons were limited by heterogeneous postoperative analgesic regimens or unequal local anesthetic dosing between study groups. Consequently, whether this technically more complex block provides meaningful benefit over simpler wound infiltration within contemporary multimodal analgesia pathways remains uncertain.

This randomized controlled trial compared ultrasound-guided transversalis fascia plane block with local wound infiltration after cesarean delivery, using an equivalent total dose of local anesthetic within a standardized multimodal analgesia protocol. We hypothesized that TFPB would reduce 24-h postoperative opioid consumption compared with LWI. The primary outcome was total opioid consumption in the first 24 h. Secondary outcomes included Numeric Rating Scale (NRS) scores, rescue analgesia requirements, and postoperative nausea and vomiting (PONV).

## METHODS

This prospective, single-blinded, randomized controlled trial was approved by the Marmara University Faculty of Medicine Ethics Committee (Approval Date: 14.05.2024, No: 09.2024.217) and registered at clinicaltrials.gov (NCT06425718) before enrollment. Written informed consent was obtained from all participants, and the study adhered to the Declaration of Helsinki. Between May and June 2024, consecutive patients meeting the eligibility criteria who were scheduled to undergo cesarean section under spinal anesthesia with American Society of Anesthesiologists (ASA) physical status II–III were invited to participate. Exclusion criteria included: refusal or withdrawal of consent, inability to give consent, allergy to local anesthetics, block area infection, chronic opioid use and inability to use the patient-controlled analgesia (PCA) system.

Randomization and blinding: Participants were randomized using a computer-generated sequence (randomizer.org). Allocation was concealed with sequentially numbered, opaque, sealed envelopes opened immediately before intervention. All blocks were performed by a single experienced anesthesiologist not involved in data collection or analysis, while LWI was performed by the surgeon. Postoperative outcomes were assessed by a blinded pain team, and patients were also blinded, with interventions concealed under standard dressings. The study followed Consolidated Standards of Reporting Trials (CONSORT) guidelines (Figure 1).

**Figure 1:**
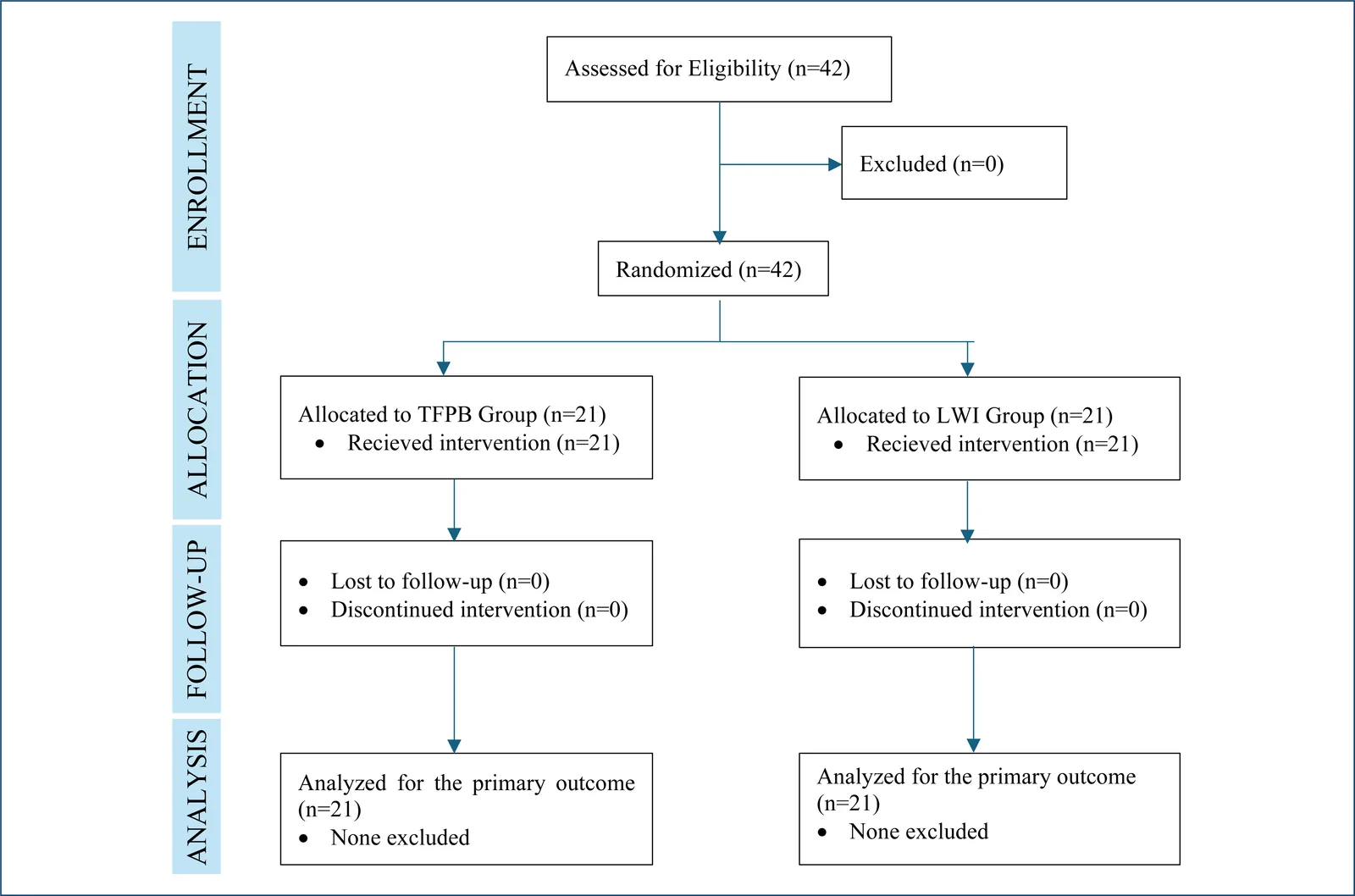
Consolidated Standards of Reporting Trials flow diagram of the study. TFPB: transversalis fascia plane block; LWI: local wound infiltration.

Intraoperative management: All patients received standardized anesthesia. Standard monitoring [electrocardiography (ECG), noninvasive blood pressure, pulse oximetry] was applied, with oxygen (4 L/min) via face mask and a 20 G intravenous (IV) cannula. Patients <150 or >150 cm received 1.8 or 2.0 mL hyperbaric bupivacaine (5 mg/mL) with 20 mcg fentanyl intrathecally, respectively. Ondansetron (4 mg IV) was given for antiemetic prophylaxis, and oxytocin was administered post-delivery per protocol.

At surgery end, in the TFPB group, a curvilinear probe (2–5 MHz Sparq Ultrasound; Philips, USA) was placed in the midaxillary line between the iliac crest and rib cage. After identifying the external oblique, internal oblique, and transversus abdominis muscles, 20 mL of 0.25% bupivacaine was injected bilaterally using an in-plane technique following saline confirmation. In the LWI group, 40 mL of 0.25% bupivacaine was infiltrated subcutaneously into the wound by the surgeon. Both groups received IV paracetamol (1 g), IV dexamethasone (8 mg), and intramuscular (IM) diclofenac (75 mg) for multimodal analgesia.

Postoperative management: All patients received IV paracetamol (1 g every 8 h) and IV patient-controlled analgesia with tramadol (5 mg/mL; no basal infusion, 1 mL bolus, 10 min lockout). If NRS >3, IM diclofenac (75 mg; max two doses/day) was administered as rescue analgesia.

In the TFPB group, sensory block was assessed after recovery from spinal anesthesia using cold and pinprick testing to confirm dermatomal involvement and exclude block failure, defined as absence of sensory changes. The block was evaluated by the anesthesiologist performing TFPB, who was not involved in data collection or analysis. Block duration was not assessed, as comparison with local wound infiltration was not feasible.

Data collection: Baseline demographic and intraoperative data were recorded. Postoperative assessments were performed at 0, 6, 12, 24, and 48 h by a pain team blinded to group allocation. At each assessment, NRS score, total tramadol consumption, rescue analgesia requirement and opioid-related adverse events were recorded.

Outcome measures: The primary outcome was 24-h total tramadol consumption. Secondary outcomes included NRS scores (0–10), rescue analgesia requirements (defined as NRS >3), and opioid-related adverse events (nausea/vomiting, urinary retention, sedation, pruritus, and respiratory depression) recorded as present or absent.

Sample size: Pilot data for sample size estimation were retrospectively obtained from institutional records of CS patients managed with the same analgesic protocol. The pilot dataset included 12 patients per group and was used solely for variance estimation and effect size approximation. The sample size was estimated using pilot data in which mean (standard deviation [SD]) tramadol consumption at 24 h postoperatively was 135.0 (51.3) mg in the control group and 85.4 (49.5) mg in the intervention group (≈37% reduction). Using G*Power 3.1 for a two-tailed Wilcoxon-Mann-Whitney test (α=0.05, power=0.80; Vargha-Delaney A≈0.76), 19 patients per group were required^9^. Allowing for 10% dropout, 21 patients per group were planned (total n=42).

Statistical analysis: Statistical analysis was performed using IBM Statistical Package for the Social Sciences (IBM SPSS) Statistics v27. Data distribution was assessed with the Shapiro-Wilk test and histograms. Continuous variables are presented as mean±SD or median [interquartile range (IQR)], as appropriate. The primary outcome (24-h opioid consumption) was non-normally distributed and analyzed using the Mann-Whitney U test with confidence intervals. Secondary continuous outcomes were analyzed similarly, while categorical variables were compared using Fisher's exact test. All tests were two-tailed, with p<0.05 considered significant.

## RESULTS

Forty-two patients were randomized and completed the study. We did not observe any block failure. The CONSORT flow diagram is shown in Figure 1.

Baseline demographic and clinical characteristics were comparable between the two groups, with no statistically significant differences in any baseline variables (Table 1). The primary outcome, total opioid consumption during the first 24 postoperative hours, did not differ significantly between the TFPB and LWI groups (median 100 [65–152] mg vs. 85 [65–135] mg, 95%CI −30.12, 51.27, p=0.554). Median opioid consumptions at all time intervals were comparable among the study groups (Table 2).

**Table 1:** Demographic data and characteristics of the participants.

		Total (n=42)	Group TFPB (n=21)	Group LWI (n=21)	p-value	Sth diff
Age (year)		31.5±6.5	32.5±6.0	30.4±6.9	0.319^a^	0.32
BMI (kg/m^2^)		30.7±4.4	29.9±3.9	31.4±4.9	0.505^a^	0.34
ASA	II	40	21 (100)	19 (90)	0.152^b^	
	III	2	0 (0)	2 (10)		

a Mann-Whitney-U test.

b Fisher's exact test. Values are presented as number of patients (%) or mean±SD. ASA: American Society of Anesthesiologists physical status; BMI: body mass index; TFPB: transversalis fascia plane block; LWI: local wound infiltration. Standardized differences are presented for continuous variables only.

**Table 2:** Comparison of total tramadol consumption during predefined postoperative time intervals between the transversalis fascia plane block and local wound infiltration groups.

IV PCA		Total (n=42)	Group		95%CI of the difference	p-value^a^
			Group TFPB (n=21)	Group LWI (n=21)		
0–6th hour	Median (Q1, Q3)	30 (20, 61.25)	45 (20, 70)	25 (17.5, 50)	-7.67, 35.77	0.193
6–12th hour	Median (Q1, Q3)	30 (15, 50)	25 (12.5, 55)	30 (20, 42.5)	-22.51, 15.37	0.528
12–24th hour	Median (Q1, Q3)	30 (20, 36.25)	30 (17.5, 37.5)	30 (22.5, 37.5)	-12.57, 12.57	0.674
24–48th hour	Median (Q1, Q3)	50 (35, 70)	40 (32.5, 75)	50 (45, 75)	-25.88, 15.88	0.332
24th hour total	Median (Q1, Q3)	90 (65, 142.5)	100 (65, 152)	85 (65, 135)	-30.12, 51.27	0.554

Summary statistics are reported as median (interquartile range). Postoperative opioid consumption is expressed in milligrams (mg). CI: confidence interval; IV PCA: intravenous patient-controlled analgesia; NRS: Numeric Rating Scale; TFPB: transversalis fascia plane block; LWI: local wound infiltration.

a Compared by Mann-Whitney-U test.

Postoperative pain scores remained low and were comparable between groups throughout the 48-h follow-up period (Table 3). Median pain scores were recorded to be <3/10 across all time intervals, indicating that postoperative pain was generally well controlled within the target range. Rescue analgesia was uncommon. In the LWI group, three patients (14.2%) required rescue analgesia, whereas no patients in the TFPB group required rescue analgesia; however, this difference was not statistically significant (p=0.072).

**Table 3:** Postoperative pain scores (Numeric Rating Scale) at predefined postoperative time points in the transversalis fascia plane block and local wound infiltration groups.

NRS		Total (n=42)	Group		95%CI of the difference	p-value^a^
			Group TFPB (n=21)	Group LWI (n=21)		
6th hour	Median (Q1, Q3)	2 (1, 2)	2 (1, 3)	2 (1, 2)	-0.56, 0.94	0.70
12th hour	Median (Q1, Q3)	1 (1, 2)	1 (1, 2)	2 (1, 2)	-0.89, 0.22	0.20
24th hour	Median (Q1, Q3)	1 (1, 2)	1 (1, 2)	2 (1, 2)	-0.84, 0.27	0.28
48th hour	Median (Q1, Q3)	1 (1, 1)	1 (0, 1)	1 (1, 1.5)	-0.67, 0.9	0.14

CI: confidence interval; NRS: Numeric Rating Scale; TFPB: transversalis fascia plane block; LWI: local wound infiltration.

a Compared by Mann-Whitney-U test.

PONV requiring medication was not observed in either group. Furthermore, no patients experienced less common opioid-related side effects such as itching, respiratory depression, or urinary retention. Additionally, no complications related to TFPB or LWI were observed.

## DISCUSSION

In this randomized trial evaluating postoperative analgesia after cesarean delivery, ultrasound-guided transversalis fascia plane block did not reduce 24-h opioid consumption compared with local wound infiltration within a multimodal analgesia protocol. Pain scores, rescue analgesia use, and opioid-related adverse events were similar. These findings suggest no clinically meaningful advantage of the more complex fascial plane block over wound infiltration.

TFPB is a deep abdominal wall block targeting the proximal ilioinguinal and iliohypogastric nerves, potentially providing broader dermatomal coverage than superficial blocks^5,10,11^. Injection beneath the transversalis fascia may allow spread toward thoracolumbar nerves before lateral branching, theoretically enhancing analgesia in lower abdominal surgery^12–14^. However, this trial showed no clinically meaningful opioid-sparing effect of TFPB when compared with LWI. Several factors may explain the lack of a statistically significant difference. Both interventions were applied within a standardized multimodal regimen including spinal anesthesia, regular paracetamol, nonsteroidal anti-inflammatory drug (NSAID) rescue analgesia, and tramadol PCA. Contemporary cesarean analgesia increasingly depends on multimodal regimens that maintain acceptable pain control while reducing opioid requirements. In this setting, baseline analgesia may already provide adequate pain relief, thereby diminishing observable differences between regional techniques. Consequently, the additional analgesic benefit of further fascial plane blocks within such pathways may be limited.

Previous studies on TFPB in cesarean delivery show heterogenous results. Compared with inactive or sham controls, TFPB reduces postoperative opioid consumption, supporting its analgesic efficacy^12,13^. However, its advantage appears less pronounced when compared with other active analgesic techniques. In a comprehensive meta-analysis on the effectiveness of local anesthetic techniques for post-CS analgesia, it was found that wound infiltration reduced total morphine consumption at hours 12 and 24 compared to inactive controls, while TFPB showed similar results to the inactive controls^14^. The only randomized trial directly comparing TFPB with wound infiltration found similar analgesic requirements, consistent with the present study^15^. Chilkoti et al. reported longer time to first analgesic request and lower NRS scores with TFPB at some time points, but pain scores remained <3 and opioid use was minimal despite the absence of adjuncts in the subarachnoid block, limiting clinical relevance. In addition, unequal local anesthetic dosing (20 vs. 40 mL, both 0.375% ropivacaine) may have biased results in favor of TFPB. In contrast, the present study standardized both volume and dose (40 mL of 0.25% bupivacaine in each group), reducing potential bias and enabling a more balanced comparison of the two techniques.

Evidence from other surgical procedures supports this interpretation. In patients undergoing iliac crest bone graft harvesting, TFPB reduced opioid consumption during the first eight postoperative hours compared with a sham block, although this benefit was not sustained thereafter^16^. In another study of inguinal hernia repair, quadratus lumborum block was associated with lower postoperative opioid consumption than TFPB^17^. These findings suggest that TFPB provides effective postoperative analgesia, but its opioid-sparing effect is more evident when compared with placebo or no regional block than with another effective regional analgesic technique.

Within an optimized multimodal cesarean analgesia pathway and with standardized local anesthetic dosing, our findings suggest that the additional benefit of complex regional techniques is limited, and LWI provides comparable analgesia with greater practicality. LWI is simpler than TFPB, requiring no ultrasound guidance or advanced expertise, has a shorter learning curve, and can be performed by the surgeon during closure. This increases its feasibility, particularly in resource-limited settings. Overall, these results support LWI as a pragmatic option within ERAS protocols, aligning with goals of reduced opioid use, early mobilization, and enhanced recovery^18^.

We observed no postoperative nausea and vomiting in either group. The traditional belief that the use of opioids contributes to nausea and vomiting has been recently questioned, as low-dose lipophilic opioids (e.g., fentanyl) used as spinal adjuvants may have antiemetic effects^19,20^. No TFPB-related complications (organ/vascular injury, infection, motor weakness) were observed. As TFPB is performed more posteriorly than transversus abdominis plane (TAP) block, and the perinephric fat lies beneath the transversalis fascia, the risk of peritoneal puncture is minimal.

A key strength of this trial is the standardization of total local anesthetic dose and volume between groups, minimizing a potential source of bias seen in previous studies where unequal dosing may have influenced outcomes. In addition, postoperative analgesia was standardized, allowing a more balanced comparison of techniques. All block procedures were performed by a single experienced anesthesiologist, reducing inter-operator variability.

The study has several limitations. The sample size, based on pilot data, may have overestimated the effect size; however, differences in opioid use were minimal and unlikely to be clinically meaningful. The lack of statistical significance should be interpreted within the context of optimized multimodal analgesia, where incremental benefits may be limited, rather than attributed solely to insufficient power. The absence of a placebo/sham group prevents assessment of absolute efficacy. The anesthesiologist who performed the TFPB also assessed block success, introducing a potential source of assessment bias, although postoperative outcomes were evaluated by blinded investigators. Finally, block duration was not evaluated beyond confirmation of success.

Future studies with larger cohorts and a sham-controlled design are needed to confirm these findings. Research should also evaluate adjuncts such as dexamethasone or dexmedetomidine to enhance the opioid-sparing effect of TFPB, while assessing their safety in postpartum patients, particularly regarding neonatal exposure via breastmilk. In addition, patient-centered outcomes, including mobility, breastfeeding comfort, and quality of recovery, should be incorporated to better define the clinical value of regional techniques in obstetric anesthesia.

## CONCLUSION

In conclusion, within a standardized multimodal cesarean analgesia protocol, ultrasound-guided TFPB did not reduce postoperative opioid consumption compared with LWI. With standardized local anesthetic dosing, the additional complexity of TFPB did not confer a clinically meaningful advantage. Both techniques provided comparable analgesia with low pain scores. Given its simplicity and practicality, LWI remains a reasonable option for routine use.

## Data Availability

The datasets generated and/or analyzed during the current study are available from the corresponding author upon reasonable request.
